# Bio‐Inspired Fractal Robust Hydrogel Catheter for Intra‐Abdominal Sepsis Management

**DOI:** 10.1002/advs.202303090

**Published:** 2023-10-11

**Authors:** Lichun Wang, Wenzhao Li, Min Li, Puxiang Lai, Chunhua Yang, Hui Wang, Bo Ma, Rongkang Huang, Yan Zu

**Affiliations:** ^1^ Department of Critical Care Medicine The Sixth Affiliated Hospital Sun Yat‐sen University Guangzhou 510655 China; ^2^ Oujiang Laboratory (Zhejiang Lab for Regenerative Medicine, Vision, and Brain Health) Wenzhou Institute University of Chinese Academy of Sciences Wenzhou Zhejiang 325001 China; ^3^ Department of Biomedical Engineering The Hong Kong Polytechnic University Hong Kong SAR 999077 China; ^4^ Department of Gastrointestinal Surgery Southern Medical University Affiliated Dongguan Shilong Peoples Hospital SSL Center Hospital Dongguan City Dongguan 523326 China; ^5^ Department of General Surgery (Colorectal Surgery) and Guangdong Provincial Key Laboratory of Colorectal and Pelvic Floor Diseases Guangdong Institute of Gastroenterology Biomedical Innovation Center The Sixth Affiliated Hospital Sun Yat‐sen University Guangzhou 510655 China; ^6^ Department of Urology The Sixth Affiliated Hospital Sun Yat‐sen University Guangzhou 510655 China

**Keywords:** bio‐inspired, biomaterials, drainage catheter, intra‐abdominal sepsis, porous hydrogel

## Abstract

To deal with intra‐abdominal sepsis, one of the major global causes of death in hospitalized patients, efficient abscess drainage is crucial. Despite decades of advances, traditional catheters have demonstrated poor drainage and absorption properties due to their simple tubular structures and their dense nonporous surface. Herein, inspired by porous sponges and fractal roots, a multifaceted hydrogel catheter with effective drainage, absorptive, and robust properties, is presented. Its unique fractal structures provide extensive internal branching and a high specific surface area for effective drainage, while the hierarchical porous structures provide a wide range of absorption capabilities. Additionally, its distinctive multi‐interpenetration network maintains robust and appropriate mechanical properties, even after absorption multiple times of liquid and mechanical disturbance, allowing for intact removal from the abdominal cavity without harm to the animal in vivo. Besides, the loaded antimicrobial peptides are capable of being released in situ to inhibit the potential for infections. In vivo experiments have demonstrated that this hydrogel catheter efficiently removes lethal abscesses and improves survival. It is believed that this innovative and practical catheter will create a future precedent for hydrogel drainage devices for more effective management of intra‐abdominal sepsis.

## Graphical abstract

Inspired by porous sponges and fractal roots, a multifaceted hydrogel catheter is developed with effective drainage, absorptive, and robust properties for intra‐abdominal sepsis management. Its fractal design ensures efficient internal branching and high surface area for drainage, while hierarchical pores offer broad absorption. Its multi‐interpenetration network maintains robust properties and loaded antimicrobial peptides can be released to prevent infections.

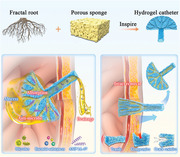

## 1 Introduction

Intra‐abdominal sepsis is highly prevalent in clinical practice, with a mortality rate as high as 29.1%.^[^ ^1^, ^2^, ^3^, ^4^, ^5^ ^]^ The effective drainage of lethal abscesses formed in the abdominal cavity through catheterization is the primary approach for treatment.^[^ ^1^, ^6^ ^]^ However, the simple tubular structures and dense nonporous surfaces of traditional catheters often lead to blockages and inadequate drainage and hinder the absorption of harmful substances produced by sepsis. Furthermore, long‐term implantation of most traditional catheters can cause severe tissue damage due to their hard‐material composition.^[^ ^7^ ^]^ Catheters with desirable properties typically include mechanical stability, efficient drainage, and toxin absorption. Despite advancements in catheter design, contemporary catheters often cannot simultaneously meet all these requirements. Hydrogels, emerging biomedical materials characterized by their soft nature and customizable shapes, show great potential in addressing the aforementioned challenges.^[^ ^8^, ^9^, ^10^, ^11^, ^12^, ^13^ ^]^ However, most hydrogels suffer from inherent weaknesses related to swelling‐weakening, making them unsuitable for in vivo applications and may even result in hazardous fragments and foreign body residues.^[^ ^14^, ^15^, ^16^ ^]^ Therefore, the development of a flexible and robust hydrogel catheter that incorporates an effective drainage structure and possesses the capacity to absorb harmful substances has remained highly anticipated for the treatment of intra‐abdominal sepsis.

**Figure 1:**
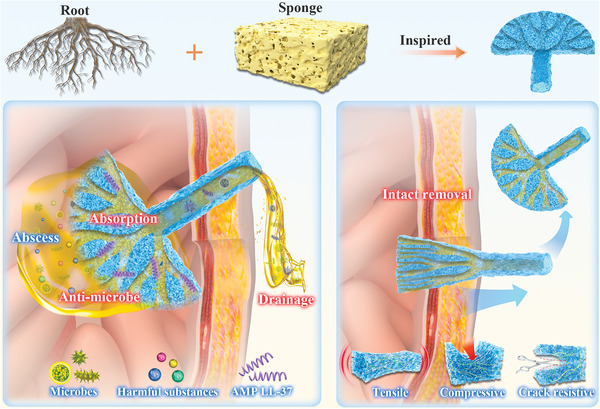
Scheme of the multi‐interpenetration network hydrogel catheter with a fractal porous structure. Inspired by the plant roots and sponges for enhanced drainage efficiency and a wide range of absorption capabilities. The catheter is loaded with antimicrobial peptide LL37 for in situ release to inhibit infection. The flexible multi‐interpenetration network exhibits desirable mechanical strength.

Herein, inspired by plant roots and sponges, we have presented a novel porous fractal multi‐interpenetration network (MIPN) hydrogel catheter for intra‐abdominal sepsis treatment, which offers effective drainage, absorptive, and enhanced robust properties (**Figure** **1**). In general, integrating both strong absorption and high mechanical properties into a hydrogel catheter applied to the abdominal cavity, a multi‐interpenetration network is a great potential strategy to provide hydrogel catheters with flexibility and robustness.^[^ ^17^, ^18^, ^19^, ^20^, ^21^ ^]^ However, many MIPN hydrogels, with strong cross‐linked structures often exhibit densely porous surfaces leading to poor absorption performance. In nature, root‐like fractal structures can enhance transport and drainage efficiency.^[^ ^22^, ^23^, ^24^ ^]^ At the same time, a sponge‐like porous structure provides a high specific surface area, enabling efficient absorption of harmful substances.^[^ ^25^, ^26^ ^]^ Therefore, our design is inspired by plant roots and sponges, aiming to integrate fractal and porous structures with a multi‐interpenetration network for hydrogel catheters, which is expected to further extend the drainage areas and reduce blockage events. However, the development of a hydrogel catheter with the integrated features mentioned above for the treatment of abdominal sepsis has not yet been reported.

In this paper, we explore the implementation of an innovative MIPN hydrogel catheter with root‐like fractal structures and sponge‐like hierarchical porous structures. Its fractal structures, precisely shaped by a 3D printing template, provide extensive internal branching and high specific surface areas.^[^ ^27^ ^]^ Thus, they result in the facilitation of drainage for abdominal abscesses. Its hierarchical porous structures, including intrinsic micropores and additional macropores created by sacrificial vaterite CaCO_3,_^[^ ^28^, ^29^ ^]^ endow the catheter with a wide range of absorption capabilities. A multi‐interpenetration network of polyacrylamide (PAAm), sodium alginate (SA), and chitosan (CS) has both desirable mechanical strength and compatible flexibility with the abdominal wall. During the invasive implantation and removal procedures, the hydrogel catheter maintains its structural integrity, even when it suffers from high swelling and mechanical disruption. Furthermore, the loaded broad‐spectrum antimicrobial peptide (AMP) LL37 releases in situ to inhibit infection progression without the abuse of anti‐infective agents.^[^ ^30^, ^31^, ^32^ ^]^ We have demonstrated that our hydrogel catheter removes lethal abscesses and improves survival in intra‐abdominal sepsis. This catheter is expected to set a precedent for hydrogel drainage devices and will encourage its widespread application for intra‐abdominal sepsis management.

## 2 Results and Discussion

### 2.1 Manufacture and Characterization of Hydrogel Catheters

**Figure 2:**
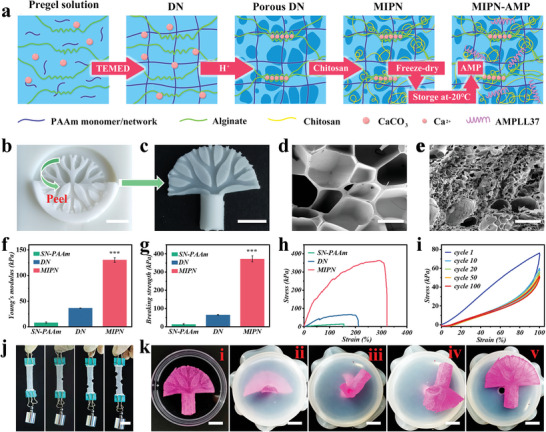
Manufacture and characterization of a hydrogel catheter. a) Fabrication process of the porous multi‐interpenetration network hydrogel. b) Fabrication of a hydrogel catheter using a 3D printing template. c) Bio‐inspired fractal hydrogel catheter. SEM images of d) DN hydrogel and e) MIPN hydrogel. f) Young's modulus and g) breaking strength of different hydrogel samples. h) Stress–strain curves and i) tensile cyclic stress‐strain curves of a variety of hydrogel samples. j) Mechanical test to monitor hydrogel crack resistance. k) Schematic representation of hydrogel removal from a small hole. Scale bars are 1 cm in (b and c), 500 µm in (d and e), 2 cm in (j), and 1 cm in (k).

In this experiment, the hydrogel catheter was manufactured through three main processes: cross‐linking in a fractal mold, sacrificial template pore‐creation, and drug loading. The fractal structure of the hydrogel catheter is designed as a multi‐level branching according to the corresponding geometry and fluid dynamics calculations (Figure S1, Supporting Information). First, a pregel solution, consisting of acrylamide (AAm), SA, and vaterite CaCO_3_ (**Figure** **2**a), was poured into the 3D‐printed fractal catheter mold (Figure 2b; Figure S1, Supporting Information). The first PAAm network was then cross‐linked spontaneously (Figure 2a). The structure of the fractal groove on the surface, formed by the 3D mold, converged into a hollow tube with an opening at the end upon demolding (Figure 2c). Subsequently, the obtained hydrogel catheter was immersed in a mixed solution of HCl and CaCl_2_, and the vaterite CaCO_3_ was sacrificed to release Ca^2+^, resulting in the generation of pores (Figure S2, Supporting Information). The second alginate network was cross‐linked by Ca^2+^ (Figure 2a). Sponge‐like macropore structures with an average diameter of 263.24 ± 149.03 µm were observed under optical microscopy and scanning electron microscopy (SEM) (Figure 2d; Figure S4, Supporting Information). Furthermore, chitosan, a polycation, penetrated the pores and the previous hydrogel network through molecular‐level interpenetration under vacuum. It was then cross‐linked with the NaOH solution to establish its own network structure. The introduction of chitosan facilitated the formation of electrostatic interactions with sodium alginate, resulting in significantly stronger bonding forces compared to the alginate‐PAAm network alone. Additionally, acrylamide (covalently cross‐linked), alginate (ionically cross‐linked), and chitosan (H‐bonding cross‐linked) interacted with each other through H‐bonding, electrostatic, and Van der Waals interactions, which endowed the hydrogel with desirable mechanical properties. The chitosan‐filled porous structure was also clearly observed under SEM (Figure 2e). These additional macropores, along with inherent micropores, together formed a hierarchical pore structure that not only facilitated the absorption of various harmful substances in the abdominal cavity but also ensured the sustained release of antimicrobial chitosan and LL37. Finally, the hydrogel catheter could be loaded with the AMP LL37 for further application (Figure 2a). The final MIPN‐AMP hydrogel or hydrogel catheter loaded with AMP (HC‐AMP) was prepared using the following proportions: 0.1 g hydrogel was evenly infiltrated by 100 µL LL37 solution (0.60 mM). Fourier transform infrared spectroscopy (FTIR) demonstrated the components of the hydrogel network and the interactions between them (Figure S3, Supporting Information).

Inside the abdominal cavity, the ability to maintain mechanical properties after absorption and swelling is a widespread and tricky challenge for the hydrogel. This prevents the intact removal of the hydrogel and can even lead to residual foreign material. Although the introduction of cross‐linking agents and adjusting their density can enhance the mechanical properties of hydrogels, a higher concentration of cross‐linking agents often results in an increased Young's modulus and a decreased breaking strength. As a consequence, the hydrogel may become brittle and inflexible, posing risks of tissue damage and the foreign body residual in vivo. The MIPN strategy we developed has a more robust network strength, and we anticipate it to solve these problems. To further demonstrate the effect, we carried out mechanical property tests. In comparison to the single network (SN) and double network (DN) hydrogel with the same composition source, our porous MIPN hydrogel exhibited the highest result for Young's modulus (130.46 ± 5.54 kPa) (Figure 2f). The breaking strength of the MIPN hydrogel also exhibited a similar trend (Figure 2g). Representative stress‐strain curves are shown in Figure 2h. Furthermore, Young's modulus and breaking strength were maintained at 37.65 ± 3.77 and 42.25 ± 4.13 kPa (Figure S5a–c, Supporting Information) even after immersion in PBS for 96 h. To simulate repeated mechanical impacts in the abdominal cavity, cyclic tensile tests were performed at 0–100% strain continuously for 100 cycles and MIPN hydrogel swelled for 96 h. This procedure resulted in a recovery ratio of 68% and still maintained its original shape (Figure 2i). We also observed that this swelled MIPN hydrogel could protect against crack formation (Figure 2j), which was a benefitted form of the sacrificial network in the hydrogel.

To simulate the mechanical damages during the removal process, the MIPN hydrogel underwent swelling in PBS to achieve a liquid perfusion‐hydrogel catheter. We observed instances where it underwent twisting, stretching, and folding, yet it could be uniformly restored to its original shape (Figure S5d, Supporting Information). In addition, the polyvinyl chloride (PVC) tube displayed its hardness properties (Figure S5e, Supporting Information). The MIPN hydrogel catheter was then removed through a 0.5 cm diameter hole while preserving its original shape without any fracture (Figure 2k; Video S1, Supporting Information). Thus, our MIPN hydrogel exhibited desirable mechanical properties which facilitated its intact removal from the abdominal cavity without harm to the animal or destruction of the hydrogel.

### 2.2 In Vitro Absorption and Drainage Capacity

**Figure 3:**
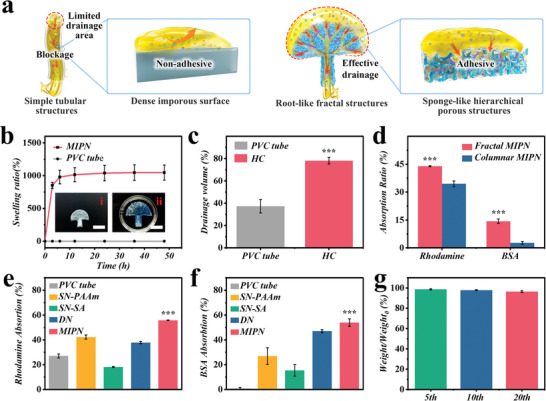
In vitro absorption and drainage capacity. a) Scheme of the superiority of absorption and drainage capacity of our hydrogel catheter compared with a traditional PVC tube. b) Swelling properties of MIPN hydrogel and a PVC tube. The scale bars are 2 cm. c) In vitro drainage capacity of hydrogel catheter compared with PVC tube. d) In vitro absorption capacity of fractal catheter compared with columnar shape. Surrogate test of cytokine absorption based on the absorption of e) BSA and f) rhodamine. g) Weight change under cycles of repeated stretching and removal from a 0.5 cm hole.

Removal of harmful substances is vital for intra‐abdominal sepsis, yet traditional catheters fall short in this area. A hierarchical porous and fractal structure provided a hydrogel catheter with distinguishable absorption and drainage capabilities, while the traditional catheters exhibited poor drainage and absorption properties due to their simple tubular structures and dense nonporous surfaces (**Figure** **3**a). First, to investigate the liquid absorption ability, we assessed the equilibrium swelling ratios of the hydrogels in PBS (Figure 3b). The MIPN hydrogel rapidly absorbed liquid within the first 6 h, then reached equilibrium after 48 h, with an absorption rate over 10 times its initial mass, and a maximum swelling ratio of 1045.97 ± 115.01%. This fulfilled the need to absorb physiological fluids in an abdominal cavity. Such a characteristic was not possessed by the PVC tube. The swelling ratio of volume change was 345.00 ± 21.53%, aligning with the trend of swelling ratio based on mass (Figure S6, Supporting Information). The designed fractal structures of the hydrogel catheter offered extensive internal branching and a high specific surface area, enhancing the efficiency in draining an abdominal abscess. In vitro experiments showed that 78% of the solution could be drained via a liquid perfusion‐hydrogel catheter while only ≈40% via the PVC tube (Figure 3c; Videos S2 and S3, Supporting Information), indicating superior drainage efficiency.

The urgent necessity for further removal of specific molecules, such as inflammatory mediators and LPS, is essential in the prevention of sepsis. This action will circumvent the excessive stimulation of the host immune system, thereby averting potential life‐threatening organ dysfunction. When contrasted with traditional clinical PVC tubes, our hydrogel catheters possess a distinctively extensive array of absorption capabilities. We showed the LPS absorption capacity by immersing the hydrogel catheters in an LPS solution for 24 h. As depicted in Figure S7 (Supporting Information), LPS absorption efficiency achieved a record high of 63.8% after 24 h. Furthermore, we studied the absorption capacity of inflammatory mediators using two model molecules. Bovine serum albumin (BSA) was utilized as a macromolecule model given that it exhibits similar chemical properties to cytokines IL‐6 and TNF‐α. Rhodamine was adopted as a minor model molecule for certain inflammatory mediators, such as malondialdehyde. As illustrated in Figure 3d–f, thanks to its porous structure, the hydrogel catheter could absorb a broader range of substances at a significantly higher rate than traditional PVC tubes and other types of hydrogels. Moreover, due to its fractal structure, the hydrogel catheter displayed superior absorption capacity compared to the same hydrogel in columnar shape (Figure 3d). In addition, the liquid perfusion‐hydrogel catheter experienced no significant mass reduction after 20 cycles of repetition–stretching and removal from a 0.5 cm hole (Figure 3g), indicating its excellent retention capacity following absorption. As a result, these fractal porous hydrogel catheters are ideally suited to prevent the generation of diverse harmful substances that might cause sepsis.

### 2.3 In Vitro Antimicrobial Activity

**Figure 4:**
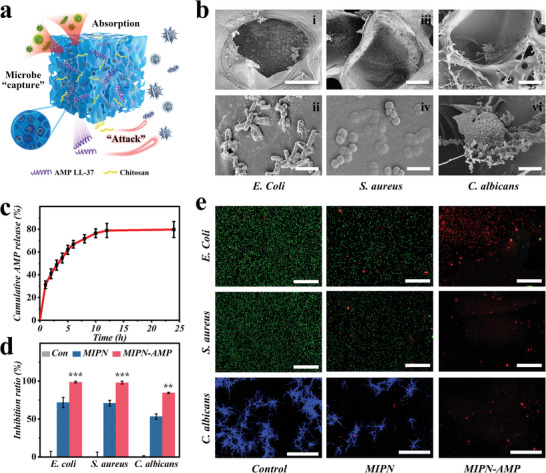
In vitro antimicrobial activity. a) Scheme of antimicrobial mechanisms of hydrogel catheter. b) SEM micrographs of pathogenic microorganisms in hydrogel pores. c) Cumulative drug release from hydrogel over time. d) Inhibition ratio of microbe in each group. e) Inverted fluorescence micrographs of microbe in each group. Scale bars in (b) are 50 µm in (i), 2.5 µm in (ii), 50 µm in (iii), 2.5 µm in (iv), 100 µm in (v), and 25 µm in (vi). Scale bars in (e) are 50 µm in *E. Coli* and *S. aureus*, and 100 µm in *C. albicans*.

Efficient elimination of microbes is critical to prevent intrabdominal sepsis (**Figure** **4**a). Regardless of drug absence, the macropores in the hierarchical pore system efficiently ensnared microbes at the micron scale. Moreover, the chitosan in the MIPN system exhibited antimicrobial characteristics. To exhibit the antimicrobial prowess of our hydrogel, we exposed the MIPN hydrogel to individual *E. coli*, *S. aureus*, and *C. albicans* solutions. The aforementioned microbes are representative of the leading gram‐negative/positive bacteria and fungi in sepsis. After a 24 h immersion in microbial solution, we noticed that the microbes were entrapped within the macropores (Figure 4b(i, iii, iv)). Additionally, a high‐magnification SEM image unveiled structural disturbances in these microbes, explicitly noticeable in the case of *E. coli* (Figure 3(ii)). This phenomenon can be ascribed to the reduction of microbial viability by the existence of pores and the antimicrobial activity of chitosan.

In addition, our porous catheter served as a drug carrier loaded with antimicrobial substances. LL37 is a multifunctional peptide of the cathelicidin family that exhibits broad‐spectrum antimicrobial activity against microbes. Accordingly, LL37 was loaded into the MIPN hydrogel catheter in this study to increase antimicrobial efficacy and improve the outcome. We monitored the AMP release rate to indicate whether the MIPN‐AMP hydrogel would continuously release as needed. The MIPN‐AMP hydrogel exhibited an initial rapid‐release period with a cumulative AMP release of 31% after the first hour and 72% after 8 h. Such an early surge of drug release is beneficial for quickly reaching an effective antimicrobial concentration. For the following 24 h, the MIPN‐AMP hydrogel displayed a smooth and sustained release process resulting in a cumulative release of 80%, with 20% of AMP retained within the hydrogel (Figure 4c). This was followed by a more gradual release imparting persistent microbial activity.

Quantitatively assessing, the antimicrobial efficacy of our developed hydrogel against the aforementioned pathogenic microorganisms was evaluated (Figure 4d,e). After interacting with the MIPN hydrogel for 8 h, 71.77% of *E. coli*, 70.90% of *S. aureus*, and 53.26% of *C. albicans* were suppressed. When co‐cultured with MIPN‐AMP hydrogels, 98.62% of *E. coli*, 98.00% of *S. aureus*, and 84.42% of *C. albicans* were inhibited, thus demonstrating the excellent antimicrobial properties of the hydrogel. The above‐stated outcomes were further corroborated by the plate‐coating technique (Figure S8, Supporting Information). A hydrogel catheter loaded with antimicrobial peptide attains potent antimicrobial impacts, serving to amplify the therapeutic effectiveness while minimizing any adverse systemic side effects.

### 2.4 In Vitro Biocompatibility

**Figure 5:**
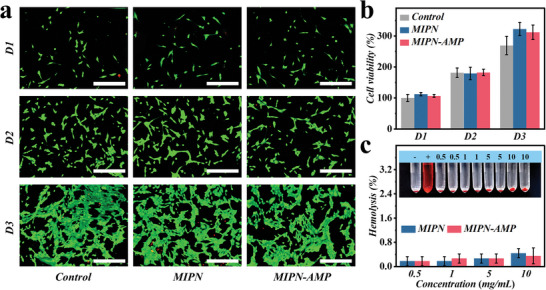
In vitro cytotoxicity and hemolysis assays. a) Live/dead staining images of NIH‐3T3 fibroblast cultures and b) Quantitative analysis by CCK‐8 assay of cell viability. c) Hemolysis assay with photograph (inset) showing erythrocytes incubated with varying amounts of hydrogel. Scale bars are 100 µm in (a).

Exceptional cytocompatibility and hemocompatibility are crucial given that the hydrogel catheters were implanted into the abdominal cavity. For the cytocompatibility test, model cell NIH‐3T3 fibroblasts were cultured in leachate derived from hydrogel catheters. Over a span of 3 days of cultivation, live/dead stained images indicated by fluorescence scanning demonstrated that NIH‐3T3 fibroblasts in both the control and experimental groups were dispersed uniformly, exhibiting normal morphology (**Figure** **5**a). Additionally, CCK‐8 assays for quantitative statistics were conducted. Over 3 days, there was a steady increase in cell viability with no discernible differences between groups. These qualitative and quantitative outcomes collectively indicate that our hydrogel catheters do not adversely affect the cells (Figure 5b). Moreover, an in vitro hemolytic experiment was utilized to assess hemocompatibility. Neither MIPN‐AMP hydrogel nor MIPN hydrogel exhibited signs of hemolysis even at concentrations as optimal as 10 mg mL^−1^ (Figure 5c). These findings confirm that our MIPN hydrogel exhibits superior biocompatibility and is suitable for biomedical applications in order to prevent sepsis.

### 2.5 In Vivo Therapeutic Efficacy of Hydrogel Catheters

**Figure 6:**
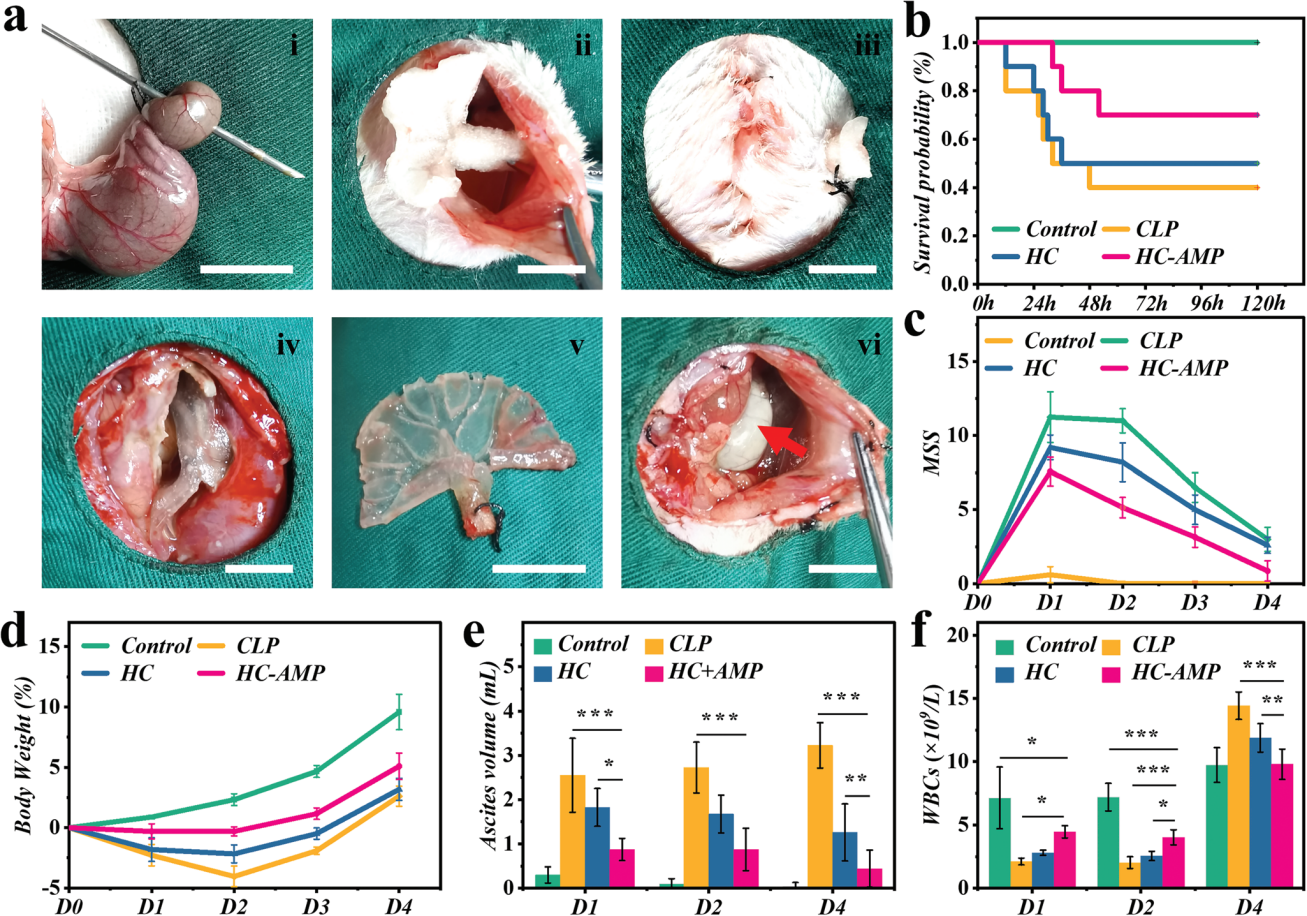
Sepsis treatments in the control, CLP, HC, and HC‐AMP groups. a) Overview of the CLP procedure and schematic for implant removal. (i) cecal puncture with a needle; (ii) hydrogel catheter implantation via abdominal wall incision; (iii) placement of hydrogel catheter and closure of abdominal incision; (iv) in vivo appearance of hydrogel catheter after 4 days of placement; (v) intact removal of hydrogel catheter from the abdominal cavity; (vi) appearance of caecum necrosis (red arrow) on the fourth postoperative day. b) Kaplan–Meier plots representing the survival probability of rats. c) MSS in each group. d) Relative change in rats’ body weight measurements. Time course measurements of e) ascites volume; f) WBC values. Rats were randomly assigned to four groups. The scale bars are 1.5 cm.

To further assess the therapeutic potency of the hydrogel catheter, a model of intra‐abdominal sepsis was employed in rats using cecal ligation and puncture (CLP) (Figure S9, Supporting Information). This procedure simulated the pathogenesis and progression of human sepsis and was conducted on two sets of rats. These rats were randomly divided into four groups: the sham laparotomy group (control group), CLP group treated with PVC tube (CLP group), hydrogel catheter (HC) group, and LL37 loaded hydrogel catheter group (HC‐AMP group). The first group of 35 rats, comprised of 5 in the control group, 10 in each of the CLP group, the HC group, and the HC‐AMP group were utilized for observation of general condition and mortality rate. Afterward, they were sacrificed to conduct WBC count, cytokine tests, and to collect ascitic fluid on the fourth postoperative day. In the second set of rats (*n* = 56), 8 were allocated to the control group, 20 to the CLP group, 16 to the HC group, and 12 to the HC‐AMP group. Every four surviving rats from each group were sacrificed to conduct WBC, and cytokine tests, and to collect ascitic fluid respectively on the first and second postoperative days. In the HC and HC‐AMP groups, the hydrogel catheters were implanted into the abdominal cavity (**Figure** **6**a(i–iii)). On the fourth postoperative day, no signs of blockage were observed in the hydrogel catheters, confirming their facilitation in drainage. Due to their exceptional mechanical properties, the hydrogel catheters were successfully removed from the abdominal cavity without any damage. Further, no significant evidence of abdominal bleeding was noticed, indicating the favorable biocompatibility and tissue‐sparing nature of our hydrogel catheter (Figure 6a(iv–vi)).

The evaluation of treatment effectiveness for sepsis necessitates an encompassing assessment that takes into account multiple factors, such as survival rates, disease condition improvement, and the management of infection source control. In Figure 6b, it was observed that the HC‐AMP group showcased the maximum survival rate at 70%, followed by the HC group at 50%. The CLP group had a survival rate of 40% in contrast. The reduced mortality exhibited in the HC‐AMP group was attributed to the drainage and absorptive properties of the hydrogel catheter, alongside the antimicrobial functionality of the loaded AMP. The severity of sepsis in rats was assessed via a murine sepsis severity (MSS) scoring system. On the first postoperative day, the MSS scores in the CLP, HC, and HC‐AMP groups were elevated compared to the control group, indicating the successful implementation of the sepsis model. However, on the second postoperative day, the HC‐AMP group demonstrated a significant enhancement in MSS scores compared to the CLP group, trailed by the HC group (Figure 6c, all *p* < 0.05). A similar pattern was observed in the measurement of rat body weight (Figure 6d). Rats suffering from intra‐abdominal sepsis experienced notable weight loss post‐CLP surgery. The mildest weight loss was identified in the HC‐AMP group, while the most drastic weight loss was detected in the CLP group (Figure 6e, all *p* < 0.05). All experimental groups developed ascites, with the maximum volumes identified in the CLP group and the minimum in the HC‐AMP group (Figure 6e). Compared to the CLP group, the HC group revealed noticeable improvements in sepsis severity scores, weight loss, and ascitic fluid volume. Particularly, the HC‐AMP group exhibited the most significant enhancements in these indicators. These outcomes indicated that our hydrogel catheter alleviates sepsis severity in CLP rats, particularly when loaded with antimicrobial peptides.

To better comprehend the influence of the hydrogel catheter on infection severity in CLP rats, WBC was assessed at various intervals post‐procedure (Figure 6f). In reaction to intra‐abdominal sepsis, all experimental groups demonstrated a downward trend in WBC counts within the initial 2 days. The HC‐AMP group showed a minor decrease, the HC group underwent a moderate decline, while the CLP group displayed a substantial drop. Subsequently, WBC counts in the HC‐AMP group returned to baseline on the fourth day, yet remained escalated in both the HC and CLP groups. Our investigations further establish that our hydrogel catheter can mitigate infection severity in intra‐abdominal sepsis, with an enhanced therapeutic efficacy when loaded with antimicrobial peptides.

**Figure 7:**
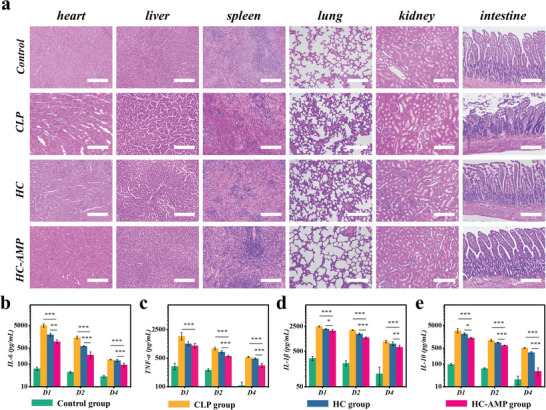
Protective efficacy of a hydrogel catheter in CLP rat model. a) Representative histological staining of major organs 24 h post‐operation. b–e) Expression of inflammatory mediators IL‐6, TNF‐α, IL‐1β, and IL‐10 detected by ELISA. The scale bars represent 200 µm.

Pathological alterations in major organs were examined using tissue histology stained with hematoxylin and eosin (**Figure** **7**a). The findings indicated that the employment of HC‐AMP substantially minimized tissue injury and tempered inflammation in the CLP rat model. The hydrogel catheter, even in the absence of antimicrobial peptides, showed a moderate abatement of these impacts compared to the CLP group (Figure 7a). Disruption, lysis, intracellular edema, and swelling in heart tissue were less marked in the HC‐AMP group. Moreover, the HC‐AMP group manifested reduced hepatocellular damage and inflammation, encompassing mild disorders of hepatic lobules and diminished infiltration of inflammatory cells into liver tissue. In a similar vein, the HC‐AMP group exhibited minimal congestion and edema in the spleen. No substantial signs of hemorrhage, alveolar congestion, or thickening were detected in the lungs of this group. In addition, the HC‐AMP group showed no obvious tubular cell injury, epithelial cell edema, or dilation of the tubular lumen in the kidney. The infiltration of inflammatory cells and shed microvilli in the intestinal lumen were notably reduced in the HC‐AMP group. These modifications suggest that the hydrogel catheter loaded with LL37 has the potential to ease tissue damage and inflammation.

During sepsis, there is a close association between the cytokine storm, immune dysregulation, and organ damage. To understand the impact of the LL37‐loaded hydrogel catheter on inflammatory conditions, we analyzed inflammatory cytokines (Figure 7b,e). Our findings indicated that IL‐6, TNF‐α, IL‐1β, and IL‐10 levels in the plasma were notably higher in all three experimental groups as opposed to the control group, with these observations being made on subsequent postoperative days. These results pointed to systemic inflammation in the CLP model. Among the three experimental groups, the HC‐AMP group displayed the lowest cytokine levels, followed by the HC group with moderate levels, meanwhile, the CLP group had the highest levels of inflammatory cytokines (Figure 7b,e, all *p* < 0.05). All inflammatory cytokines peaked on the first postoperative day and decreased over the following days. These outcomes are consistent with prior research. The observed alterations in cytokine levels correlated closely with the pathological damage observed in rats. This correlation signifies a strong connection to the drainage, absorption, and antimicrobial properties possessed by the LL37‐loaded hydrogel catheter.

## 3 Conclusion

In conclusion, inspired by sponges and plant roots, we have successfully prepared a novel flexible yet robust hydrogel catheter with fractal and porous structures for intra‐abdominal sepsis management. The MIPN hydrogel is constituted by PAAm, SA, and chitosan networks, exhibiting both desirable strength and compatible flexibility with an abdominal wall, even after absorbing over 10 times the liquid of its initial mass. The bio‐inspired fractal structures, shaped by 3D printing templates, provide extensive internal branching and a high specific surface area for effective drainage, while the hierarchical porous structures afford a wide range of absorption. In addition, the loaded antimicrobial peptides are released appropriately for effective antimicrobial therapy. The hydrogel possesses favorable biocompatibility to meet the requirements of intraperitoneal implantation, and its outstanding mechanical properties allow for intact removal from the abdominal cavity without harm to the animal or destruction of the hydrogel in vivo. We have demonstrated that our integrated hydrogel catheter can significantly reduce levels of inflammation, prevent multiple organ injury, and improve survival in septic animals. Thus, we believe that this hydrogel catheter can provide a novel and promising treatment for intra‐abdominal sepsis in clinics and create an unprecedented hydrogel drainage device for future use in intra‐abdominal sepsis management.

## 4 Experimental Section

The Experimental section is available in Supporting Information.

## Conflict of Interest

The authors declare no conflict of interest.

## Author Contributions

L.C.W. and W.Z.L. contributed equally to this work. H. W., P.X.L, M.L., C. H. Y., B.M., R. K. H., and Y. Z. conceived the idea and designed the experiment. L.C.W. and W.Z.L. conducted experiments and data analysis. L.C.W., W.Z.L., R. K. H., and Y.Z. wrote the manuscript.

## Supporting information

Supporting Information

Supplemental Video 1

Supplemental Video 2

Supplemental Video 3

## Data Availability

The data that support the findings of this study are available in the supplementary material of this article.
