# Supplementary material for: Bio‐Inspired Fractal Robust Hydrogel Catheter for Intra‐Abdominal Sepsis Management
Source: Adv Sci (Weinh). 2023 Oct 11;10(32):2303090. doi: 10.1002/advs.202303090 (PMC10646267; doi:10.1002/advs.202303090)
Supplement: Supplementary file 1 — Supporting Information [file ADVS-10-2303090-s003.pdf]

ADVANCED SCIENCE
Open Access

Supporting Information

for *Adv. Sci.*, DOI 10.1002/advs.202303090

Bio-Inspired Fractal Robust Hydrogel Catheter for Intra-Abdominal Sepsis Management

Lichun Wang, Wenzhao Li, Min Li, Puxiang Lai, Chunhua Yang*, Hui Wang, Bo Ma*, Rongkang Huang* and Yan Zu**

Supplementary Information

Bio-inspired fractal robust hydrogel catheter for intra-abdominal sepsis management

Lichun Wang[1], Wenzhao Li[2,3], Min Li[4, *], Puxiang Lai[3], Chunhua Yang[1, *], Hui Wang[5], Bo Ma[6, *], Rongkang Huang[5, *], Yan Zu[2, *]

[1] Department of Critical Care Medicine, The Sixth Affiliated Hospital, Sun Yat-sen University, Guangzhou, 510655, China

[2] Oujiang Laboratory (Zhejiang Lab for Regenerative Medicine, Vision and Brain Health), Wenzhou Institute, University of Chinese Academy of Sciences, Wenzhou, Zhejiang 325001, China

[3] Department of Biomedical Engineering, The Hong Kong Polytechnic University, Hong Kong SAR, 999077, China

[4] Department of Gastrointestinal Surgery, Southern Medical University, Affiliated Dongguan Shilong Peoples Hospital, SSL Center Hospital Dongguan City, Dongguan 523326, China

[5] Department of General Surgery (Colorectal Surgery) and Guangdong Provincial Key Laboratory of Colorectal and Pelvic Floor Diseases, Guangdong Institute of Gastroenterology, Biomedical Innovation Center, The Sixth Affiliated Hospital, Sun Yat-sen University, Guangzhou 510655, China

[6] Department of Urology, The Sixth Affiliated Hospital, Sun Yat-sen University, Guangzhou 510655, China

* Corresponding authors

Emails: zuyan@ucas.ac.cn (Y. Zu); huangrk3@mail.sysu.edu.cn (R. Huang);
mabo3@mail.sysu.edu.cn (B. Ma); drlimin@dingtalk.com (M. Li);
ychhua@mail.sysu.edu.cn (C. Yang)

Fractal Structures and Their Hydrodynamic Properties

The design is based on a comprehensive analysis of four main factors: fractal geometry, fluid dynamics, physiological environment, and hydrogel fabrication process. Firstly, it is inspired by the fractal structures of natural efficient fluid transport systems such as plant roots, human blood vessels, hence, we used a fractal tree model to construct the basic channel paradigm of the hydrogel catheter **(Fig. S1a)**. In simple terms, a fractal tree consists of a "trunk" and many "branches", each branch looks like the tree itself, thus creating a self-similar object. Using affine transformation iteration, in the form of $\vec{t}_{n+1} = f(\vec{t}_n) = A\vec{t}_n + \vec{h}$, the branches from the previous iteration are used as the trunk for the current iteration. This results in a hierarchical fractal structure. The parameters *r*, ϕ and *b* are used to define a strict fractal tree. Where *r* is the scaling ratio for each iteration, ϕ is the rotation angle of the branches relative to the trunk during iteration, and b is the number of branches.

To normalize the fractal geometric structure, we set $r = \sqrt{2}/2$, which is the common silver ratio constant in fractal geometry. In each individual bundle of branches, we denote the one with the largest angle with the positive direction of the x-axis as the first branch, Then we set the angle between the first and the final branch in each bundle of fractal structure to be $\theta = 2\pi/3$ and make the bundle of branches symmetric about their trunk, so that the shape of the catheter is basically "umbrella-like", meeting the requirements of implantation in vivo. Then we set the angle between the *m*'th branch and the first branch to satisfy $\phi = (m/b) * \theta$, so as to ensure the uniform distribution of branch angles. In the x-y plane, we define the trunk $\vec{t}_0$ as a standard vector parallel to the y-axis, that is $\vec{t}_0 = \begin{bmatrix} 0 \\ 1 \end{bmatrix}$, with its starting point at the origin O (0,0). To ensure the existence of a fractal structure, we should have $b \geq 2$, and enumerate 2, 3, 4 and other positive integers as potential values for *b*. In this case, for each iteration from the trunk $\vec{t}_n$ to the branches $\vec{t}_{n+1}$ of the fractal tree, the corresponding affine transformation is

$$\tau(\mathrm{m},\mathrm{n}) = \vec{\mathrm{t}}_{\mathrm{n}+1} = \vec{\mathrm{t}}_{\mathrm{n}} + \mathrm{r}\begin{bmatrix} \cos((\pi/3)-\phi) & -\sin((\pi/3)-\phi) \\ \sin((\pi/3)-\phi) & \cos((\pi/3)-\phi) \end{bmatrix}\vec{\mathrm{t}}_{\mathrm{n}} .$$

To illustrate, we take b=3 and iteration order n=2 as an example. The shape of the fractal tree defined in this project is shown in the Fig. S1b.

Then, fluid dynamics calculations were used to further obtain the appropriate geometric structure. According to the spatial structure of the abdominal cavity in vivo, taking, $|\vec{\mathrm{t}}_0| = 2\ \mathrm{cm}$ and the cross-sectional area as $2\ \mathrm{mm}^2$ an example, each line segment in the fractal tree was regarded as a cylindrical fluid channel and given a spatial geometric structure. To ensure the comparability of the structures, it was stipulated that the sum of the cross-sectional areas of all branches after each iteration was equal to the cross-sectional area of the trunk.

After calculating by the mass equation

$$\frac{\partial(u)}{\partial x} + \frac{\partial(v)}{\partial y} + \frac{\partial(w)}{\partial z} = 0$$

and momentum equation

$$\begin{cases} \frac{\partial(u)}{\partial t} = -\frac{1}{\rho}\frac{\partial p}{\partial x} - \left(\frac{\partial\left(u^2\right)}{\partial x} + \frac{\partial(uv)}{\partial y} + \frac{\partial(uw)}{\partial z}\right) + \frac{1}{\rho}\left(\frac{\partial \tau_{xx}}{\partial x} + \frac{\partial \tau_{yzx}}{\partial y} + \frac{\partial \tau_{zxi}}{\partial z}\right) \\ \frac{\partial(v)}{\partial t} = -\frac{1}{\rho}\frac{\partial p}{\partial y} - \left(\frac{\partial(vu)}{\partial x} + \frac{\partial\left(v^2\right)}{\partial y} + \frac{\partial(vw)}{\partial z}\right) + \frac{1}{\rho}\left(\frac{\partial \tau_{xy}}{\partial x} + \frac{\partial \tau_{yy}}{\partial y} + \frac{\partial \tau_{zy}}{\partial z}\right) \\ \frac{\partial(w)}{\partial t} = -\frac{1}{\rho}\frac{\partial p}{\partial z} - \left(\frac{\partial(wu)}{\partial x} + \frac{\partial(wv)}{\partial y} + \frac{\partial\left(w^2\right)}{\partial z}\right) + \frac{1}{\rho}\left(\frac{\partial \tau_{zz}}{\partial x} + \frac{\partial \tau_{yz}}{\partial y} + \frac{\partial \tau_{zz}}{\partial z}\right) \end{cases}$$

with Catia, we obtained the velocity and pressure distribution under various fractal geometric shapes Fig. S1c-h. Under the premise of keeping the pressure through the exit of trunk $\vec{\mathrm{t}}_0$ constant, as the iteration order *n* or the number of branches *b* increased, flow resistances in the channel are increased. Therefore, to avoid excessive resistance, we limited $\mathrm{n}=2$ and $\mathrm{b} \in [2,3,4]$ in this stage. In addition, after geometric calculation, when $\mathrm{b} \geq 4$ and $\mathrm{n} \geq 2$, the channels intersected. According to the fluid dynamics calculation, the fluid interfered with each other in the channels, resulting in a

significant reduction in the velocity of the branches, which could not drain normally **(Fig. S1e and h)**. Therefore, we discarded $b=4$. For the case of $b=2$, the shortest distance between the entrances of the two adjacent bundles was $\sqrt{6}$ cm, and the distance between the adjacent entrances of the same bundle was $\sqrt{3}$ cm, which was unevenly distributed in space **(Fig. S1c and f)**. This uneven spatial distribution of fluid inlets, combined with a small number of inlets and a large flow rate at each inlet, results in that it can only centrally absorb fluid from a few directions, which is not uniform and robust. For $b=3$, these distances were $(\sqrt{2}\text{ -}1)$ cm and 1 cm respectively **(Fig. S1d and g)**. Its multiple inlets can evenly absorb fluid in multiple directions, the flow velocity distribution is reasonable, and it also has a relatively good low flow resistance. Therefore, we finally chose $b=3$ as the basis of the geometric shape. To make the shape smooth and regular to meet the preparation and application of biomedical hydrogels, we obtained the spatial structure used in this project by semi-self-similar third iteration (to prevent additional channel crossing or excessive flow resistance, we did not use complete self-similarity in the third iteration), chamfering and other spatial smoothing operations with appropriate trimming.

In the actual experiment, the fractal structures of our hydrogel catheter provide extensive internal branching and high specific surface areas. Thus, they result in the facilitation of drainage for abdominal abscesses. In vitro experiments showed that 78% of the solution could be drained via a hydrogel catheter but only approximately 40% via the PVC tube, which demonstrated better drainage efficiency of the fractal structure. In addition, due to the flexibility of 3D printing and free radical polymerization in the hydrogel preparation process in our work, the hydrogel catheters can also be further adapted to various more precise scenarios. It is expected that artificial intelligence and other new technologies can be used to screen customized fractal structures for sepsis patients in different situations with high throughput, achieving more effective and precise applications.

Materials and Methods

Materials

Sodium alginate (low viscosity), sodium alginate (median viscosity), rhodamine, and fluorescent brightener 28 were purchased from Sigma-Aldrich, USA. Acrylamide (AAm), calcium chloride anhydrous ($CaCl_2$), potassium persulfate (KPS), chitosan (CS), and tetraethyl methylenediamine (TEMED) were from Macklin, Shanghai, China. Calcium carbonate ($CaCO_3$), and bovine serum albumin fraction V (BSA) were bought from Aladdin, Shanghai, China. N, N′-methylene bisacrylamide (BIS), PBS, and ELISA kits (IL-6, TNF-α, IL-1β, IL-10) were obtained from Solarbio, Beijing, China. LL37 was bought from MedChemExpress, China. Rhodamine-conjugated LL37 was bought from China Peptides Co., Ltd., Shanghai China. NIH 3T3 was purchased from the Chinese Academy of Sciences, Shanghai, China. Dulbeccos modified eagle medium, trypsin, fetal bovine serum, and Penicillin/Streptomycin were provided by Gibco, USA. Live/dead cell viability kit, cell counting kit-8 (CCK8), and bicinchoninic acid protein assay kit was obtained from Thermo Fisher Scientific, USA. *E. coli* (ATCC8739), *S. aureus* (ATCC9144), and *C. albicans* (ATCC10231) were derived from Shanghai Bioresource Collection Center. SYTO Green and propidium iodide were provided by KeyGen Bio-Tech Co., Ltd., Jiangsu, China. The limulus amebocyte lysate endotoxin kit was bought from Xiamen Bioendo Technology Co., Ltd.

Characterization

All the hydrogels and vaterite calcium carbonate were observed by scanning electron microscope (SU8010, Hitachi, Japan). The FTIR spectra were measured on a spectrometer (TENSOR II, Bruker, Germany). The mechanical tests were carried out using a universal material testing machine (5944, Instron, USA). The absorption spectrum of the solution was evaluated by a microplate reader (Varioskan LUX, ThermoFisher, USA). Images capture was acquired on a stereomicroscope (SZX2-ILLTQ, Olympus, Japan) and an inverted epifluorescence microscope (Axio Vert.A1, Zeiss, Germany).

Preparation of Hydrogels

For the SN-SA hydrogel, sodium alginate of low viscosity (1%) and sodium alginate of median viscosity (1%) were dissolved uniformly in deionized water as a pregel solution, and using calcium chloride (5%) as the cross-linking agent, then left until complete cross-linked.

For the SN-PAAm hydrogel, AAM (15%), BIS (0.25%), and KPS (2.5%) were dissolved uniformly in deionized water as a pregel solution. Then, TEMED (1 μL mL^{-1}) was added to complete the cross-linking process.

For the DN hydrogel, sodium alginate of low viscosity (1%), sodium alginate of median viscosity (1%), AAM (15%), BIS (0.25%), KPS (2.5%) were dissolved uniformly in deionized water as pregel solution. TEMED (1 μL mL^{-1}) was added to initiate the hydrogel cross-linking. Then the obtained hydrogels were maintained in the calcium chloride solution (5%) until complete cross-linking.

For MIPN hydrogel, additional sacrificial template pore-creating was applied. $CaCO_3$ (0.05 g mL^{-1}) was homogenously dissolved and dispersed in the above DN pregel solution. TEMED (1 μL mL^{-1}) was added into the pregel solution and then poured into dishes (for films) or 3D-printed molds (for catheters). They were left until complete cross-linking, and after that, immersed in the calcium chloride (5%) and diluted hydrochloric acid (1.75%) mixture, then washed thoroughly in PBS to remove the residual hydrochloric acid. In the next step, they were soaked in CS solution (1%) in a vacuum until the pores filled with CS completely, then the hydrogel was frozen-dried. Sodium hydroxide solution (100 μL, 0.045 mg mL^{-1}) was added in 0.1 g hydrogel as cross-linkers to enhance the triple networks.

To prepare the final drug-loaded MIPN-AMP hydrogel or HC-AMP, 0.1 g hydrogel was evenly infiltrated in 100 μL LL37 solution (0.60 mM).

Mechanical Tests

Hydrogel films of SN-PAAm, DN, and MIPN with a thickness of about 2.00 mm were used for the mechanical test. Breaking strength and breaking strain were recorded until fracture. Young's modulus was calculated by the stress/strain ratio at the fracture point.

Cyclic tensile tests were conducted by 100 cycles of loading-unloading. Each experiment group were performed in 3 independent experiments.

Swelling Performance

Each MIPN hydrogel was soaked in the PBS solution to achieve an equilibrium of swelling. Equations were used to calculate the swelling ratio.

$$\text{Swelling ratio} = (W_h\text{-}W_0) / W_0 \times 100\%$$

"W_h" was the weight of swollen hydrogel at a given time point, "W_0" was the initial weight of the hydrogel.

***In Vitro* Drainage and Absorption Experiments**

For the comparison of drainage efficacy between HC and PVC tubes. HC was soaked in PBS for 48 hours. The distal opening of the HC and PVC tube (~ 2 mm inner diameter) were connected to a 20 mL syringe, respectively. The injection equipment was left in the dish (5.5 mm diameter filled with 6 mL water) then slowly withdrawn, and the remaining liquid was recorded for calculation.

To investigate the absorption capacity of different hydrogels, hydrogels of SN-PAAm, SN-SA, DN, and MIPN were prepared as films with a thickness of about 2.00 mm. All the hydrogels mentioned above were frozen-dried for absorption capacity testing. The above hydrogels (0.1667 g each) and the polyvinyl chloride tube (0.1667 g) were respectively immersed into 5 mL BSA solution (1 mg mL^{-1}) and Rhodamine solution (50 μg mL^{-1}) for 48 hours. The tube and hydrogels were taken out from the buffer, and the remaining liquid was recorded for the absorption rate calculation. BSA concentration was determined using BCA assay with BSA as standard.

$$\text{Absorption ratio } \% = 100\%\text{-}(C_0 \times 5\text{ mL- } C_{48} \times V_{48})/C_0 \times 5\text{ mL} \times 100\%$$

"C_{48}" was the concentration at 48 hours, C_0 was the concentration at 0 hour.

To compare the absorption capacity of MIPN hydrogels with different shapes at the first 1-hour, columnar MIPN hydrogel with about 1 cm diameter and fractal MIPN hydrogel with a thickness of about 2.00 mm were prepared. The procedure above was repeated, with soaking times of 1 hour.

For the LPS absorption experiment, MIPN hydrogel (0.0667 g) was immersed in 2 mL LPS solution (1EU mL^{-1}) for 24 hours. After that, MIPN hydrogels were taken out from the buffer, and the remaining liquid was recorded for the absorption rate calculation. LPS was detected by limulus amebocyte lysate endotoxin kit.

$$\text{LPS absorption ratio } \% = 100\%-(C_0\times 2\text{mL}- C_{24}\times V_{24})/C_0\times 2\text{ mL} \times 100\%$$

"C_{24}" was the concentration at 24 hours, C_0 was the concentration at 0 hours.

In Vitro Drug Release Tests

To obtain a more accurate concentration of LL37, rhodamine-conjugated LL37 was used as a substitute for drug release tests. 0.1667 g MIPN hydrogel was evenly infiltrated with 166.7 µL rhodamine-conjugated LL37 (0.60 mM) and then immersed in 5 mL PBS solution at 37 °C. At the predetermined time, the fluid was completely removed and replaced with fresh 5mL PBS. The cumulative amount of LL37 released was calculated based on standard curves and performed in 6 independent experiments.

$$\text{Cumulative release ratio } \%= \frac{\sum_1^n C_i V_i}{m_{drug}}$$

"Ci" was the concentration of the sampled. "V_i" was the volume of i the sampled. "m" was the mass of the drug.

In Vitro Antimicrobial Experiments

To assess the microbial trapping capacity of the MIPN, the 0.0333g hydrogel was soaked in 2 mL microbial liquid (OD_{600} = 0.5) in 12-well plates and cultured at 37 °C for 24 h. The hydrogels were then taken out and fixed by glutaraldehyde solution (2.5%) for 30 min, followed by rinsing three times with PBS, and then processed for SEM observation.

To understand the antimicrobial activity of MIPN and MIPN-AMP. *E. coli, S. aureus, and C. albican* liquid (OD_{600} = 0.1) were diluted 1:10. For the control groups, 2mL of the above liquid was cultured in 12-well plates. For MIPN and MIPN-AMP groups, MIPN hydrogel (0.0333g) and MIPN-AMP hydrogel (0.0333g) were put into 2 mL of the diluted microbial liquid and cultured in 12-well plates, respectively. *E. coli* and *S.*

aureus were cultured for 8 hours, while *C. albican* was cultured for 16 hours at 37 °C. Antimicrobial activity was determined according to the OD_{600} in each group. Three independent samples from each group were analyzed. Meanwhile, *E. coli* and *S. aureus* were coated and incubated on agar plates at 1:1000 dilution, *C. albican* was incubated at a 1:100 dilution. After that, live bacteria were stained green with SYTO, *C. albican* was stained blue with fluorescent brightener 28, and dead microbe was stained red by PI. Images captured were acquired by fluorescence microscopy.

In Vitro Cytocompatibility Assay

NIH-3T3 cells were cultured in 96-well plates for 3 days. For MIPN and MIPN-AMP groups, the hydrogels were soaked in the medium at the ratio of 0.0333 g mL^{-1} for 24 hours to obtain leachate. Then the filtered medium was used for NIH-3T3 cell culture. In parallel, NIH-3T3 cell was cultured in medium as a control group. CCK-8 assay and live/dead staining were performed at different culturing times (D1, D2, and D3), and image capture was acquired.

In Vitro Hemocompatibility Assay

A hemolysis assay was conducted by using rat red blood cells (RBCs). The RBCs were washed three times with 1×PBS (3000 RPM, 10 minutes), and erythrocyte suspension (1% v/v) was prepared in PBS. The MIPN and MIPN-AMP hydrogels were incubated with the above erythrocyte suspension at 37 °C for 3 hours, in the ratio of 0.5, 1, 5, and 10 mg mL^{-1}. After that, erythrocyte suspension was centrifuged for 10 min at 3000 RPM, then absorbance in the supernatant was measured at OD_{540}. RBCs dispersed in deionized water (1%, v/v) were prepared for positive control, and erythrocyte suspension (1%, v/v) in PBS was used as the negative control. All the groups were conducted in three independent experiments.

In Vivo Animal Experiment

Sepsis was induced by CLP in male SD rats (9 weeks), supplied by SPF (Beijing) Biotechnology Co., Ltd. All the animals' experiments were approved by the Animal

Ethics Committee of Guangzhou Huateng Biomedical Technology Co., Ltd. In this study, we performant two batches of rats. Rats were randomly assigned to 4 groups: 1) control group: sham laparotomy; 2) CLP group: ligation and puncture, implantation with PVC; 3) HC group: ligation and puncture, implantation with hydrogel catheter; 4) HC-AMP group: ligation and puncture, implantation with LL37 loaded hydrogel catheter. In this study, we performed two batches of rats. The first 35 rats, 5 in the control group, 10 in the CLP group, 10 in the HC group, and 10 in the HC-AMP group were used to observe the rats' general condition and mortality rate, finally they were sacrificed for WBC count, cytokines tests, and ascitic fluid collection on the fourth days after the operation. The second batch of rats (n = 56) were randomly assigned to 4 groups as mentioned above, 8 in the control group, 20 in the CLP group, 16 in the HC group, and 12 the HC-AMP group. In the second batch rats, every 4 survival rats in each group were sacrificed for WBC, cytokines tests, and ascitic fluid collection respectively on the first and second days after the operation.

CLP procedures were performed as followings. First, a median incision (~3 cm) was made in the abdominal wall. Second, the cecum was exposed, dissected the mesentery, and ligated at approximately 1 cm from the distal end with 3-0 silk suture. Third, the cecum was punctured with a 16-gauge needle and lightly squeezed until a small amount of stool was extruded. Fourth, the cecum was returned to the abdominal cavity. In the CLP, HC, HC-AMP group, a PVC tube with about 2 mm diameter, the hydrogel catheter, and LL37 loaded hydrogel catheter was implanted into the abdominal cavity near the punctured site respectively. Hydrogel catheter or PVC tube was implanted into the abdominal wall as followings. The hemostatic forceps was inserted into the midline incision on the abdominal wall. The tip of the forceps was tunneled through the abdominal wall 1cm away from the midline incision and pressed against the skin. An incision was made in the abdominal wall at the tip of the forceps using a scalpel, then the forceps was pushed through the incision. A new forceps outside the abdominal wall was grasped by the forceps and its tip is inserted 2 cm into the abdominal cavity, while releasing the original forceps. The distal end of the hollow tube of our hydrogel catheter or PVC tube was grasped by the new forceps from the incision, which was then pulled

out of the abdominal cavity and fixed to the skin. And the hydrogel catheter with fractal groove structure was placed in the abdominal cavity around the punctured cecum. Finally, the abdominal skin was closed. Each of the hydrogel catheters in this study was approximately 0.1667 g loaded with 166.7 μL LL37 (0.60 mM). All rats received injections of 30 mL kg^{-1} normal saline through tail intravenous injection after the operation. In the control group, rats received laboratory without cecum puncture and ligation. The animals were kept in their home cage and warmed by a heating lamp until they recovered from anesthesia. Rats were provided with food and water at the bottom of the cage.

Histology Analysis and Cytokines Analysis

At 24 hours, 48 hours, and 96 hours after surgery, rats were sacrificed following the established protocol. Blood was collected from sham-operated and CLP-induced septic rats by cardiac puncture under anesthesia. Plasma was stored for cytokine analysis. Blood parameters were measured by Automated Hematology Analyzer (Sysmex-xs-500i, Japan). Cytokines from plasma were measured by ELISA. Ascites was collected and measured. Heart, liver, spleen, lung, kidney, and intestine were harvested 24 hours after the operation, fixed in 4% paraformaldehyde for histologic study. Histological analysis was performed by Wuhan Servicebio Technology Co., Ltd., China.

Statistical Analysis

Statistical analysis was conducted by SPSS software (Version 22.0, IBM Corp., Armonk, NY, USA). Data expressed are expressed as mean ± SD. Differences between groups were compared and analyzed using the one-way analysis of variance (ANOVA). A p-value < 0.05 was considered statistically significant. ***: $p < 0.001$, **: $p < 0.01$, and *: $p < 0.05$.

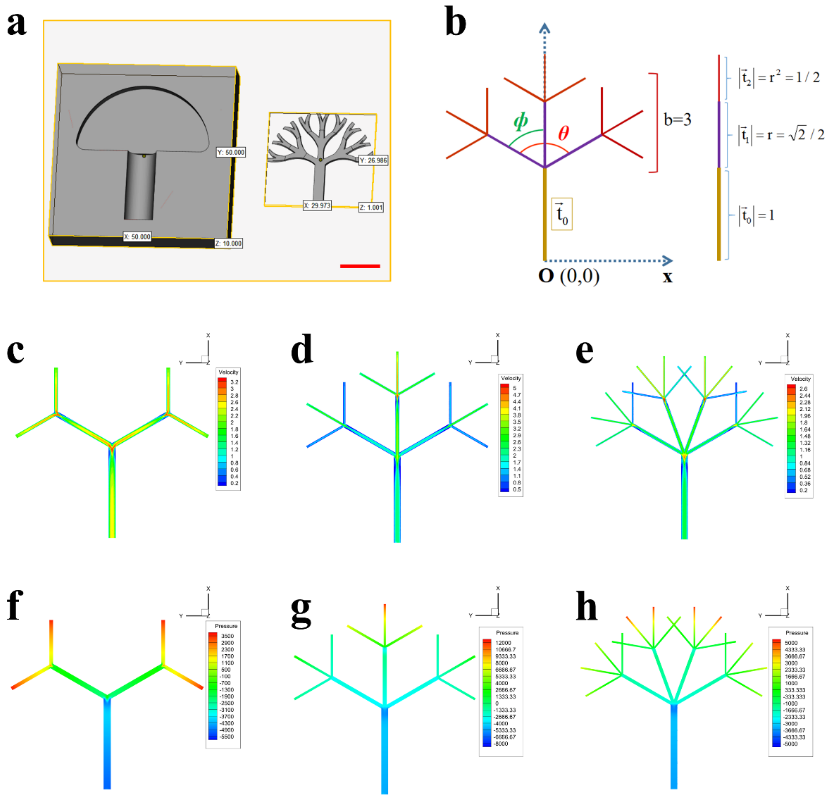

Figure S1. Fractal structures and their hydrodynamic properties. (a) 3D printed fractal catheter mold. The scale bar is 1 cm. (b) Construction and main parameters of the fractal tree. (c-e) Flow velocity simulation when (c) b=2, (d) b=3 and (e) b=4. (f-h) Pressure simulation when (f) b=2, (g) b=3, and (h) b=4.

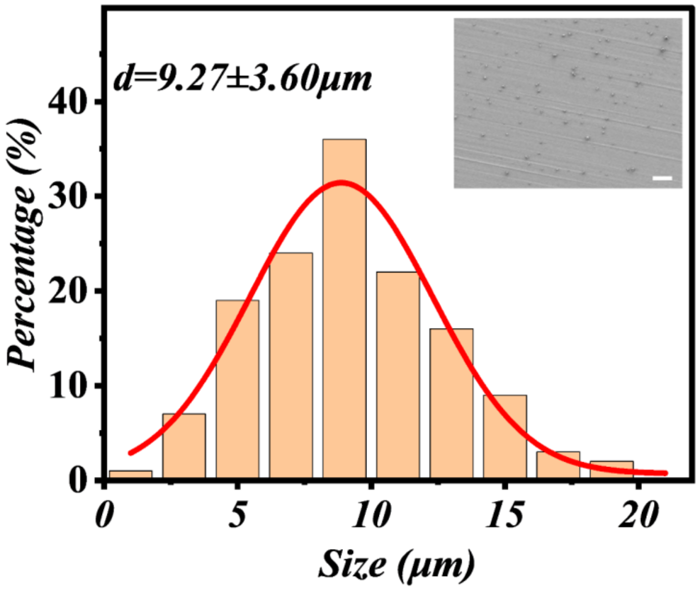

Figure S2. Particle size distribution of vaterite $CaCO_3$. The scale bar is 100 µm.

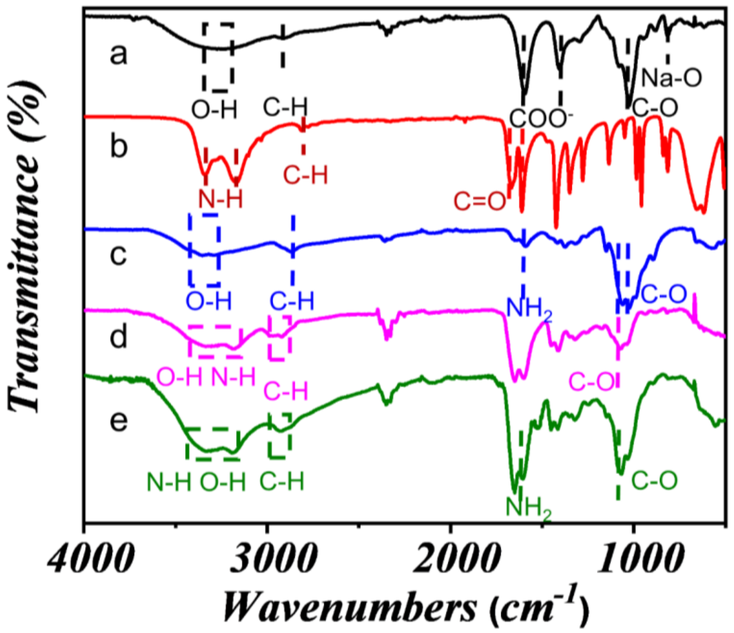

Figure S3. FTIR spectra of hydrogels cross-linked with (a) sodium alginate (SA), (b) acrylamide (AAm), (c) chitosan (CS), (d) acrylamide + sodium alginate (DN), and (e) acrylamide + sodium alginate + chitosan (MIPN).

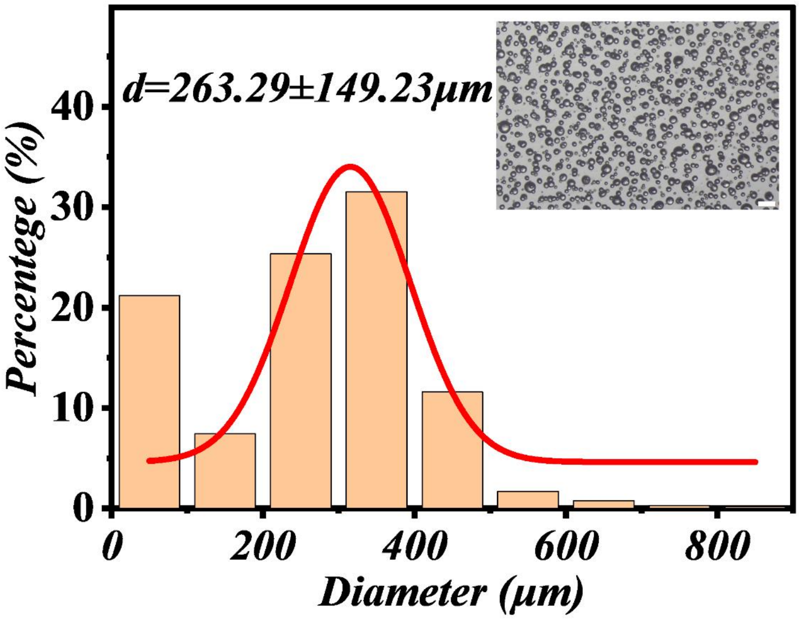

Figure S4. Pore size distribution of the hydrogel. The scale bar is 500 μm.

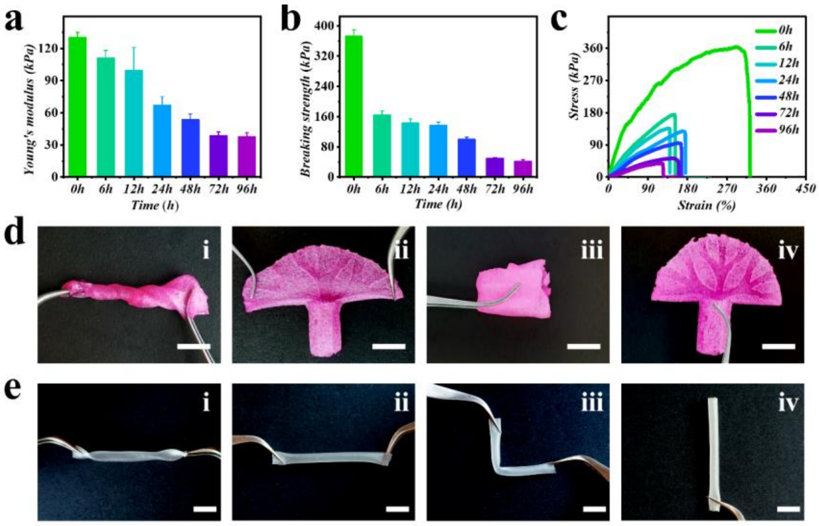

Figure S5. Tensile property analysis of the hydrogel. Time-course analysis of (a) Young's modulus, (b) Breaking strength, and (c) Stress-strain of the hydrogel. (d) Photographs of the hydrogel catheter under (i) twisted, (ii) stretch, and (iii) envelope strain; and (iv) the hydrogel after soaking in PBS for 96 hours. (e) Photographs of the PVC tube (i) twisted, (ii) stretch, and (iii) envelope strain; and (iv) the PVC tube after soaking in PBS for 96 hours. The scale bars are 1 cm.

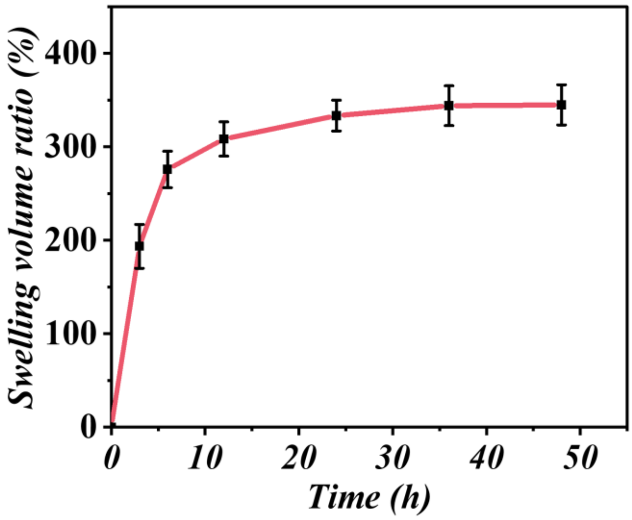

Figure S6 Swelling ratio of volume change of the MIPN hydrogel.

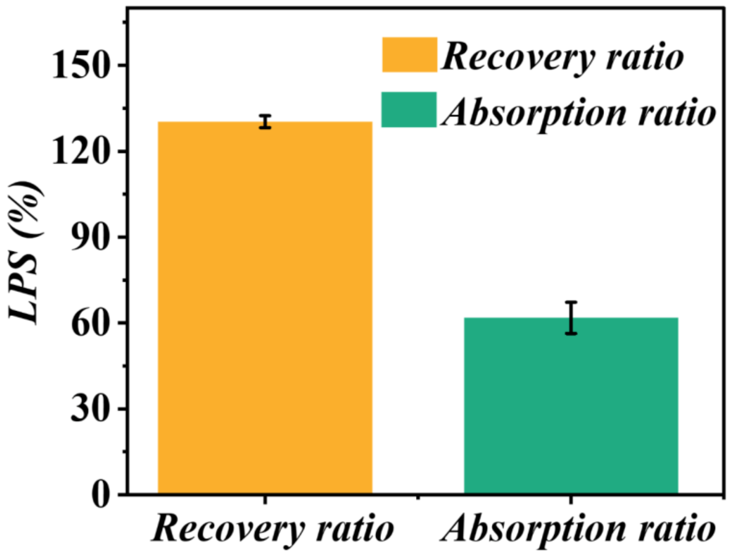

Figure S7. LPS absorption ratio of the MIPN hydrogel.

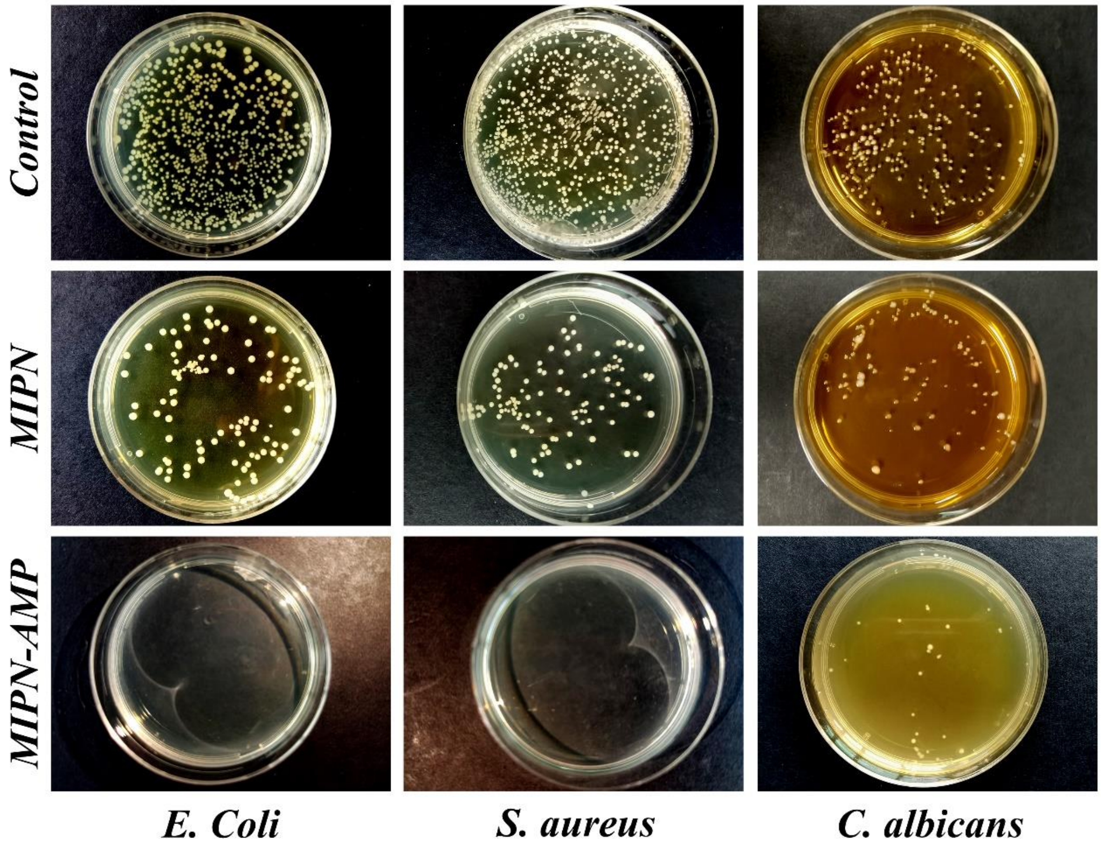

Figure S8. The panel compares the growth of *E. Coli, S. aureus*, and *C. albicans* colonies on agar culture plates in the control, MIPN, and MIPN-AMP groups.

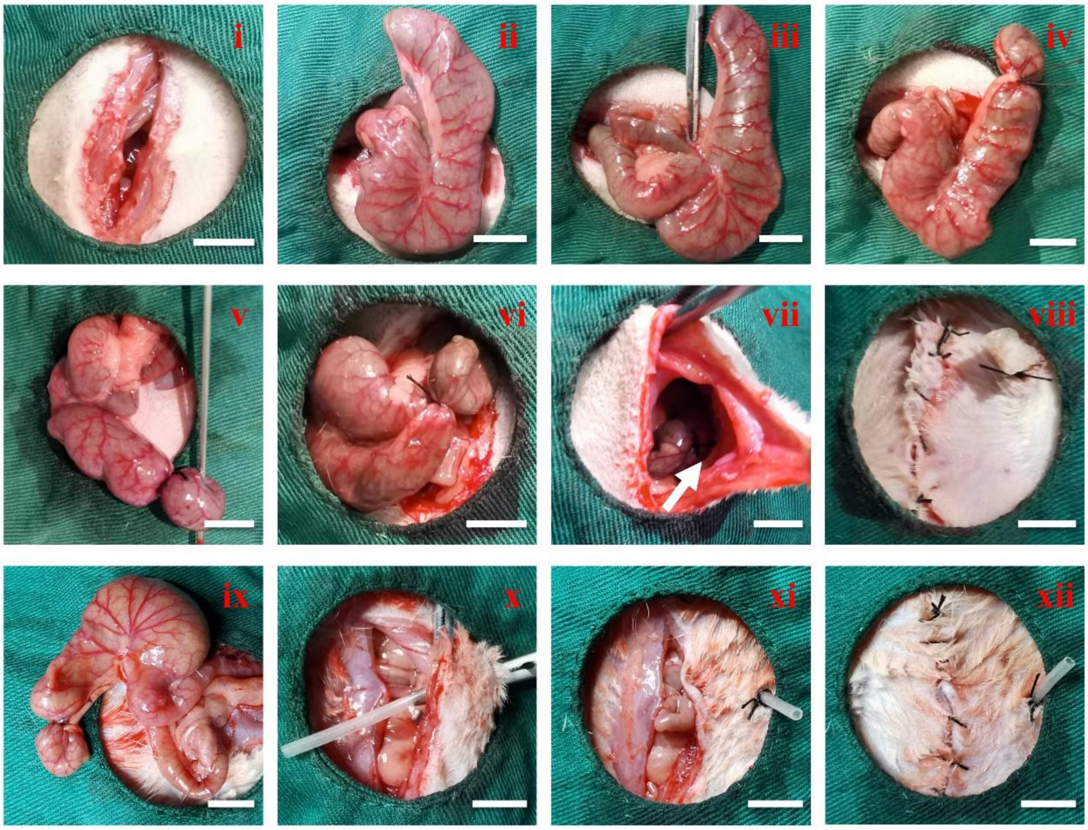

Figure S9. Cecal ligature and puncture surgical procedure (i-iv), and subsequent implantation of hydrogel (v-viii) or PVC tube (ix-xii). i: median incision; ii: cecum exposed; iii: dissected the mesentery; iv: cecum ligation; v: punctured with a needle; vi: stool extruded; vii: HC or HC-AMP implantation, white arrow points to HC or HC-AMP; viii: abdominal suture; ix: cecal ligation and perforation in CLP group; x and xi: PVC implantation; xii: abdominal suture in CLP group. The scale bars are 1 cm.
